# Fear of compassion from others explains the relation between borderline personality disorder symptoms and ineffective conflict resolution strategies among patients with substance use disorders

**DOI:** 10.1186/s40479-022-00207-8

**Published:** 2022-12-26

**Authors:** Kim L. Gratz, Warner Myntti, Adam J. D. Mann, Ariana G. Vidaña, Matthew T. Tull

**Affiliations:** 1 Department of Psychology, University of Toledo, 2801 West Bancroft Street, Toledo, OH 43606 USA; 2 Department of Psychology, Eastern Michigan University, Ypsilanti, MI USA

**Keywords:** Borderline personality disorder, Substance use disorders, Conflict, Romantic relationships, Conflict resolution, Fear of compassion

## Abstract

**Background:**

Borderline personality disorder (BPD) pathology is common among patients with substance use disorders (SUDs) and associated with a variety of negative outcomes, including worse SUD outcomes. One particularly relevant outcome with links to substance use problems that is likely to be elevated among SUD patients with BPD symptoms is ineffective conflict resolution strategies in romantic relationships. However, no research to date has examined the relation of BPD pathology to strategies for managing conflict in romantic relationships among patients with SUDs, or the factors that may increase the use of ineffective strategies within this population. Thus, this study examined the relations of BPD symptoms to ineffective responses to romantic relationship conflict surrounding substance use among residential patients with SUDs, as well as the explanatory roles of fear of compassion from and for others in these relations.

**Methods:**

Patients in a community-based correctional SUD residential treatment facility (*N* = 93) completed questionnaires, including a measure of BPD symptoms, fear of compassion from and for others, and strategies for responding to conflict surrounding substance use in romantic relationships.

**Results:**

Fear of compassion from others accounted for significant variance in the relations of BPD symptoms to the ineffective conflict resolution strategies of reactivity, domination, and submission, whereas fear of compassion for others only accounted for significant variance in the relation between BPD symptoms and the strategy of separation (which is not always ineffective).

**Conclusions:**

Together, findings suggest that it is fear of compassion from others (vs. fear of compassion for others) that explains the relation between BPD symptoms and ineffective responses to romantic relationship conflict surrounding substance use among SUD patients. Findings highlight the potential utility of interventions aimed at reducing fears of compassion and increasing comfort with and tolerance of compassion from both others and oneself among SUD patients with BPD symptoms in order to strengthen relationships and reduce risk for relapse.

## Background

Borderline personality disorder (BPD) is a serious mental health problem characterized by pervasive dysfunction across emotional, behavioral, and interpersonal domains and associated with severe functional impairment, substantial mental and physical disability, and heightened risk for a variety of self-destructive and health-compromising behaviors [1–3]. Although BPD is found at rates of 1–6% in the general population [1, 4–6], it is far more common among inpatient psychiatric populations (where rates of BPD range from 15 to 20%) [7–9], especially substance use disorder (SUD) populations. Indeed, evidence suggests that BPD pathology is particularly common among patients with SUDs, with the rate of BPD among individuals with a SUD estimated to be 22.1% and the rate among inpatients with a SUD estimated to be 26.7% [10].

Notably, the presence of BPD pathology among individuals with a SUD is associated with greater dysfunction and a variety of negative outcomes, including higher rates of suicidal and nonsuicidal self-injurious behaviors [11], higher levels of risky behaviors [12], greater psychiatric severity [13], greater physical health problems [12], poorer psychological health [12], greater substance use severity [12, 14, 15], and poorer SUD treatment outcomes [13, 16]. Although limited research has examined the relation of BPD pathology to interpersonal difficulties in particular within SUD populations, extensive evidence highlighting the centrality of interpersonal dysfunction to BPD [17] suggests that the presence of BPD symptoms among SUD patients is likely to relate to greater interpersonal difficulties within this population as well. Given that interpersonal relationships are theorized to play a key role in substance use outcomes [18–20], with supportive relationships protecting against relapse [21–23] and interpersonal difficulties and conflict increasing risk for substance use problems and relapse [24–26], identifying factors that may increase the risk of interpersonal difficulties among individuals with SUDs has great clinical and public health significance. In particular, given evidence that romantic relationship difficulties may be especially relevant to SUD outcomes (with research finding a unique link between conflict within romantic relationships specifically [vs. conflict with relatives and friends] and substance use problems [26]), research focused on identifying factors associated with romantic relationship difficulties among individuals with SUDs is needed.

BPD pathology is a particularly relevant factor to examine in this regard, given both its elevated levels among SUD populations and its strong links to interpersonal difficulties, including difficulties in romantic relationships. Indeed, although interpersonal dysfunction is considered a hallmark feature of BPD pathology [17] that can be observed across a variety of relationships (including parent-child relationships, work relationships, and friendships) [27–29], it may be most evident in the context of romantic relationships [30], which are often characterized by instability, ineffective communication, and conflict [31–35]. More specifically, in addition to being linked to romantic relationship dissatisfaction, problems, and conflict in general [33, 36, 37], BPD pathology is associated with ineffective communication and conflict resolution strategies in particular [38]. For example, BPD symptoms are associated with greater moment-to-moment dominant behaviors during romantic couples’ conversations in the laboratory [38], and a BPD diagnosis is associated with more ineffective communication behaviors in romantic relationships, both in general [31] and as observed in the laboratory [35, 38, 39].

Notably, how individuals respond to conflict in romantic relationships, particularly conflict surrounding their substance use, may have important implications for recovery or relapse among individuals with a SUD. Specifically, ineffective conflict resolution strategies can erode relationship satisfaction and increase relationship distress [40–43], increasing the risk for substance use or relapse [19, 44, 45]. Thus, it is imperative to identify factors that may increase use of ineffective strategies for managing romantic relationship conflict among SUD patients with BPD pathology. Such research has the potential to identify promising targets for interventions aimed at improving relationship satisfaction and decreasing risk for relapse among at-risk individuals with a SUD.

In identifying factors that may account for the relation between BPD pathology and ineffective strategies for managing conflict in romantic relationships, the fear of compassion from and for others warrants particular consideration. Theorized to motivate evolutionarily-adaptive cooperative and caring behaviors in the context of relationships [46], compassion may be defined as “a sensitivity to suffering in self and others with a commitment to try to alleviate and prevent it” [47]. Although considered adaptive for interpersonal relationships (as well as one’s relationship to the self) [47, 48], individuals vary in their ability and motivation to develop compassion, with some individuals experiencing fears of compassion both received from others (and directed toward the self) and felt for others [48]. These fears are theorized to be more common among certain clinical populations, with both theoretical and clinical literature linking fears of compassion from and for others to psychopathology and insecure attachment [48, 49]. Of particular relevance to the present study, not only is BPD pathology strongly associated with insecure attachment [50, 51], research has found elevated levels of fears of compassion both from and for others among individuals with BPD pathology (relative to individuals without psychopathology) [52].

Notably, fears of compassion from and for others may be particularly relevant to the strategies individuals use to manage conflict, as such fears may relate to both decreased use of nonjudgmental, affiliative, responsive, and compromise-based strategies that are more likely to mitigate conflict and increased use of avoidance, withdrawal, punitive, aggressive, submissive, or dominant strategies that are more likely to exacerbate conflict [48, 53–59]. Indeed, although limited research has examined the relation between fears of compassion and specific strategies for managing romantic relationship conflict, fear of compassion for the self has been associated with interpersonal problems [60], and fears of compassion from and for others have been negatively associated with empathy toward others [49]. Conversely, greater compassion for others (which is inversely related to the fear of compassion for others) has been linked to lower conflict [47] and more affiliative goals for interpersonal interactions [53, 61]. Finally, the related construct of mindfulness (which is theorized to be central to self-compassion, as both require nonjudgmental self-awareness and a willingness to experience and embrace potentially painful internal experiences [62, 63]) has been positively associated with effective/constructive strategies for responding to conflict and negatively associated with ineffective/destructive conflict strategies [40, 64].

Given the clinical and public health significance of identifying factors associated with romantic relationship conflict among individuals with SUDs, this study examined the relations of BPD symptoms and fears of compassion from and for others to ineffective responses to romantic relationship conflict surrounding substance use among residential patients with SUDs. Specifically, this study examined the extent to which fears of compassion account for the relations of BPD symptoms to ineffective conflict resolution strategies. We hypothesized that BPD symptoms and fears of compassion from and for others would be positively associated with ineffective strategies for managing romantic relationship conflict surrounding substance use. We also hypothesized that fears of compassion from and for others would account for significant variance in the relations of BPD symptoms to ineffective conflict resolution strategies.

## Methods

### Participants

Participants were 93 adult patients (39.8% female; 60.2% male) in a community-based correctional SUD residential treatment facility in Ohio. Patients receiving treatment in this facility are felony offenders referred through the court system and required to complete alcohol and drug testing to ensure abstinence prior to entering the facility. The treatment program is comprised of three stages of SUD treatment focused on increasing independence, facilitating integration into the community, relapse prevention, mental health, and skill development (e.g., coping and social skills, parenting). As the stages of treatment progress, patients are given more autonomy and are eventually allowed to leave the center for employment opportunities and family visits. Inclusion criteria for the current study included (1) 18 years of age or older, (2) fluent English speaker, and (3) currently in a romantic relationship for at least 6 months.

Participants in this study ranged in age from 19 to 57 years (*mean* age = 32.61 ± 7.76). With regard to racial/ethnic background, 20.4% of participants identified as a member of a racial/ethnic minoritized group, including Black/African-American (12.9%), Latinx (6.4%), Native American (1.1%), and other racial/ethnic minoritized group (1.1%); the remaining 79.6% identified as White. Most participants reported a low annual household income, with 54.4% reporting an annual household income of less than $10,000 and 10% reporting an annual household income of $10,000 to $20,000. With regard to educational attainment, 30.1% reported not completing high school, 53.8% received a high school diploma or GED, 10.8% completed some college or technical school, and 5.4% received a college degree. Most participants were unemployed (87.1%), with 11.8% working full-time and 1.1% working part-time. The mean relationship duration of participants’ current romantic relationship was 59.38 ± 57.00 months (range = 6 to 252 months). The primary substances of choice in the month prior to treatment or involvement with the legal system for participants in this study were methamphetamine (30.1%), heroin (25.8%), alcohol (21.5%), cocaine (20.4%), and other opioids or analgesics (18.3%).

### Measures

The McLean Screening Instrument for Borderline Personality Disorder (MSI-BPD) [65] was used to assess BPD symptoms. The MSI-BPD is a 10-item self-report measure of the presence versus absence of DSM BPD criteria. Participants are asked to indicate if each item (e.g., “Have you been extremely moody?”; “Have any of your closest relationships been troubled by a lot of arguments or repeated breakups?”) applies to them by responding yes (scored as 1) or no (scored as 0). Items are summed to create a continuous BPD symptom variable reflecting the overall number of endorsed BPD symptoms (*α* = .86 in this sample). The MSI-BPD has been widely used in both community [66] and clinical [67] samples. MSI-BPD scores have been found to demonstrate good reliability and construct and concurrent validity, as well as strong convergent validity with other well-established measures of BPD features [65, 67, 68].

The Fears of Compassion Scale (FOCS) [48] was used to measure participants’ fears of receiving and giving compassion. The FOCS is a 28-item self-report measure assessing fears of compassion across three domains: fear of compassion for self (15 items; e.g., “I worry that if I start to develop compassion for myself I will become dependent on it”), fear of compassion for others (10 items; e.g., “People will take advantage of you if you are too forgiving and compassionate”), and fear of compassion from others (13 items; e.g., “Feelings of kindness from others are somehow frightening”). Participants are asked to respond using a 5-point Likert-type scale ranging from 0 (*don’t agree at all*) to 4 (*completely agree*), with higher scores indicating greater fears of compassion. Given the focus of this study on fears of compassion involving others (vs. the self), only the subscales assessing fears of compassion from others and for others were included in the present study (αs = 91 and .88, respectively, in this sample). Support has been provided for the construct validity of the FOCS and its subscales, with scores on the FOCS subscales (including the two subscales used in this study) demonstrating positive associations with psychopathology and self-criticism and negative associations [48, 49, 69] with mindfulness and self-compassion [48, 49]. Moreover, scores on the FOCS have been found to distinguish between therapists and college students and between individuals with BPD and without psychopathology in meaningful and expected directions (i.e., lower scores among therapists and higher scores among individuals with BPD) [48, 52].

The Romantic Partner Conflict Scale (RPCS) [43] was used to assess strategies for responding to conflict in romantic relationships. The RPCS asks participants to think of a significant conflict they’ve had with their romantic partner recently and then indicate the extent to which each possible way of responding to conflict applies to them on a 5-point Likert-type scale (0 = *strongly disagree with statement,* 4 = *strongly agree with statement*). For the purposes of this study, instructions were modified to ask participants to think about a typical conflict they have with their current romantic partner about substance use in particular. The RPCS has six subscales: compromise (14 items; e.g., “My partner and I negotiate to resolve our disagreements”), avoidance (3 items; e.g., “I avoid disagreements with my partner”), interactional reactivity (6 items; e.g., “When my partner and I disagree, we argue loudly”), separation (5 items; e.g., “When we have a conflict, we separate but expect to deal with it later”), domination (6 items; e.g., “I try to take control when we argue”), and submission (5 items; e.g., “Sometimes I agree with my partner so the conflict will end”). One of these subscales assesses an effective/constructive strategy for responding to conflict (i.e., compromise) and the other five assess ineffective/destructive or potentially ineffective strategies. Specifically, whereas compromise is considered an effective strategy and domination, submission, and reactivity are considered ineffective or maladaptive strategies [43, 70], separation and avoidance have been conceptualized as more or less effective depending on the context (with some researchers considering separation to be adaptive and avoidance to be maladaptive [70] and others considering both strategies to vary in effectiveness depending on the circumstances surrounding their use [43]).

RPCS scores have been found to demonstrate adequate test-retest reliability and construct and criterion validity, including significant associations in expected directions with another measure of ineffective and effective conflict resolution strategies [43]. Likewise, evidence for the convergent and divergent validity of RPCS scores with measures of relationship satisfaction, communication, respect, and adaptive versus maladaptive types of passion has been provided [43, 70]. Given the focus of this study on the factors associated with ineffective strategies for responding to romantic relationship conflict, the current study focused on only the five subscales assessing ineffective or potentially ineffective conflict strategies (αs > .80 in this sample).

A self-report version of the Addiction Severity Index (ASI) [71] was used to assess current romantic relationship status (including length of the current relationship), as well as participants’ primary substances of choice in the month prior to treatment or involvement with the legal system. The ASI assesses seven potential problem areas among individuals who use substances, including medical status, employment/support status, alcohol/drugs, legal status, family history, family/social relationships, and psychiatric status. This study used the modules assessing family/social relationships and alcohol/drugs. Items assessing current relationship status and length of the current relationship were used to determine eligibility for inclusion in the current study. The item assessing participants’ primary substances of choice was used to characterize the sample.

### Procedure

The university’s Institutional Review Board and the Executive Vice President of Operations and Executive Director of the treatment facility reviewed and approved all study procedures. To be eligible to participate in the larger study from which these data were drawn, participants were required to be ≥18 years of age and speak English. Treatment facility staff identified eligible participants who were then given information about the study by a member of the research team. Following provision of written informed consent, participants completed a series of questionnaires. Participants were compensated for their time.

### Analysis plan

After computing descriptive statistics for the primary variables of interest, correlation analyses were conducted to examine interrelations among the primary variables of interest. To identify covariates for primary analyses, associations between relevant demographic and relationship variables (i.e., age, racial/ethnic background, sex assigned at birth, and length of current romantic relationship) and the outcome variables of interest were examined using correlation analyses. Variables found to be significantly correlated with the outcome variables were included as covariates in primary analyses [72]. Finally, the PROCESS (version 3.5) macro for SPSS (Model 4) [73] was used to examine the indirect relations of BPD symptoms to potentially ineffective conflict resolution strategies through fears of compassion from and for others. This model allowed us to examine whether fears of compassion accounted for significant variance in the relations between BPD symptoms and the use of potentially ineffective conflict resolution strategies. Indirect relations were evaluated using bias-corrected 95% confidence intervals based on 5000 bootstrap samples [73].

## Results

### Preliminary analyses

Descriptive statistics for and correlations among the primary variables of interest are presented in Table 1. BPD symptoms were significantly positively correlated with fears of compassion for and from others, as well as three of the strategies for responding to romantic relationship conflict, including reactivity, domination, and separation. Likewise, fear of compassion for others was significantly positively associated with reactivity, separation, and domination, and fear of compassion from others was significantly positively associated with reactivity, separation, domination, and submission.

**Table 1:** Correlations among and Descriptive Statistics for Primary Variables of Interest (*N* = 93)

Variables	1	2	3	4	5	6	7	8
1. BPD symptoms	–							
2. FOC for others	.26^*^	–						
3. FOC from others	.34^**^	.77^***^	–					
4. Avoidance	.00	−.03	.07	–				
5. Interactional reactivity	.22^*^	.31^**^	.40^***^	−.04	–			
6. Separation	.32^**^	.45^***^	.35^**^	−.03	.36^***^	–		
7. Domination	.33^**^	.21^*^	.34^**^	−.17	.71^***^	.34^**^	–	
8. Submission	.12	.20	.33^**^	.21^*^	.52^***^	.21^*^	.45^***^	–
*M*	5.50	20.32	22.44	8.73	11.35	10.69	9.28	9.10
*SD*	3.20	9.40	11.78	2.98	8.57	5.70	7.38	5.74

Note. BPD

Borderline personality disorder, *FOC* Fear of compassion. Range of possible scores: BPD symptoms (0–10); fear of compassion for others (0–40); fear of compassion from others (0–52); avoidance (0–12); interactional reactivity (0–24); separation (0–20); domination (0–24); submission (0–20)

*p < .05. **p < .01

### Identification of covariates

Results of analyses examining correlations between relevant demographic and relationship characteristics and the outcome variables revealed significant negative associations of age with both domination (*r* = −.23, *p* = .028) and submission (*r* = −.28, *p* = .007). All other correlations between potential covariates and outcomes variables were not significant (*r*s < |.18|, *ps* > .085). Thus, age was included as a covariate in primary analyses involving domination and submission.

### Primary analyses

Results of analyses examining the indirect relations of BPD symptoms to potentially ineffective conflict resolution strategies through fears of compassion from and for others are presented in Table 2. Given that BPD symptoms and fears of compassion for and from others were not significantly associated with the conflict resolution strategy of avoidance, this strategy was not examined.

**Table 2:** Models Examining the Explanatory Role of Fears of Compassion For and From Others in the Relations of BPD Symptoms to Conflict Resolution Strategies (*N* = 93)

Independent Variable (IV)	Explanatory Variable (EV)	Dependent Variable (DV)	Effect of IV on EV		Effect of EV on DV		Direct Effect		Indirect Effect			Total Effect	
			*a* (*p*)	*SE*	*b* (*p*)	*SE*	*c’*(*p*)	*SE*	*a x b*	*SE*	95% CI	*c*(*p*)	*SE*
BPD symptoms	FOC for others	Separation	.759 (.012)	.297	.260 (.004)	.087	.408 (.022)	.174	.197	.109	.032, .452	.572 (.002)	.177
	FOC from others		1.262 (.001)	.362	−.026 (.714)	.071			−.033	.103	−.257, .157		
BPD symptoms	FOC for others	Interactional Reactivity	.759 (.012)	.297	.015 (.915)	.138	.251 (.366)	.276	.011	.131	−.272, .272	.587 (.035)	.274
	FOC from others		1.262 (.001)	.362	.258 (.025)	.113			.3.25	.185	.015, .728		
BPD symptoms	FOC for others	Domination	.713 (.032)	.327	−.093 (.432)	.118	.455 (.079)	.256	−.066	.113	−.328, .129	.645 (.012)	.250
	FOC from others		1.190 (.004)	.399	.216 (.028)	.097			.257	.155	.005, .604		
BPD symptoms	FOC for others	Submission	.713 (.032)	.327	−.079 (.399)	.093	−.157 (.437)	.200	−.056	.096	−.288, .108	.024 (.904)	.199
	FOC from others		1.190 (.004)	.399	.199 (.010)	.076			.237	.129	.025, .537		

Note. BPD

Borderline personality disorder, *FOC* Fear of compassion

Analyses revealed significant indirect relations of BPD symptoms to interactional reactivity, domination, and submission through fear of compassion from others, and to separation through fear of compassion for others.

## Discussion

This study sought to examine the relations of BPD symptoms to ineffective responses to romantic relationship conflict surrounding substance use among residential patients with SUDs, as well as the explanatory roles of fear of compassion from and for others in these relations. Given that how individuals respond to conflict in romantic relationships may have important implications for recovery and relapse among individuals with SUDs [40–45], identifying factors that may increase the use of ineffective conflict resolution strategies among SUD patients with BPD pathology has the potential to highlight promising targets for interventions aimed at decreasing risk for relapse among this at-risk population. Providing partial support for study hypotheses, at a zero-order level, BPD symptoms were significantly associated with three of the strategies for responding to romantic relationship conflict examined here, including interactional reactivity, domination, and separation. These findings add to the literature on the negative outcomes associated with BPD pathology among individuals with a SUD [11, 12, 16], extending the extant research in this area to negative interpersonal outcomes as well.

Specifically, these findings suggest that BPD symptoms among patients with SUDs are associated with the greater use of two conflict resolution strategies considered to be ineffective and destructive to relationships: reactivity and domination [43, 69, 74]. Given the association between romantic relationship problems and negative substance use outcomes [26, 44, 45], these findings highlight a potential mechanism (i.e., ineffective conflict resolution strategies) that may explain the association between BPD pathology and worse SUD outcomes among SUD patients [13, 16]. Findings of the greater use of reactivity and domination among SUD patients with greater BPD symptoms are consistent with past research highlighting the relation of BPD pathology to both interpersonal reactivity in general and domination in particular in the context of romantic relationships [39, 75]. Results of this study also extend extant research on the conflict resolution strategies associated with BPD by providing support for a relation between BPD symptoms and the strategy of separation. Notably, unlike the strategies of reactivity and domination, separation is not considered to be an inherently problematic conflict resolution strategy, as its effectiveness is thought to vary depending on the context and the nature of the interaction [43, 70]. Thus, these findings suggest that BPD pathology may be related to a variety of conflict resolution strategies that vary in their effectiveness.

Providing partial support for study hypotheses, fear of compassion from others accounted for significant variance in the relations of BPD symptoms to the ineffective conflict resolution strategies of reactivity, domination, and submission (all of which are considered destructive to relationships [43, 70]). Conversely, fear of compassion for others only accounted for significant variance in the relation between BPD symptoms and separation (a strategy that is not always ineffective). Together, these findings suggest that it is fear of compassion from others (vs. fear of compassion for others) that explains the relation between BPD symptoms and ineffective responses to romantic relationship conflict surrounding substance use. Although further research is needed to clarify why this is the case, it is possible that the fear of compassion from others in particular may drive behaviors intended to minimize the likelihood of receiving compassionate responses from one’s romantic partner in an effort to protect oneself from the fears and perceived threats associated with positive evaluations from others or experiences of closeness or affiliative emotions [2, 49, 76, 77]. Indeed, BPD pathology has been linked to both the fear of positive evaluation [2, 77] and the fear of positive emotions [78], both of which may prompt the avoidance of affiliative emotions and related experiences [47, 77–79]. Although understandable as a self-protective mechanism, these behaviors are likely to have unintended negative consequences, increasing relationship distress/dissatisfaction and, ultimately, risk for relapse among individuals with substance use problems.

In the context of BPD pathology in particular, it is possible that the use of ineffective conflict resolution strategies to avoid affiliative emotions and related experiences of vulnerability or closeness is driven (in part) by efforts to protect oneself from the pain associated with the inevitable cessation of positive emotions. For example, affective contrast theory [80] suggests that the impact of an emotional experience is dependent on the extent to which it contrasts with a previous emotional state, with studies finding that negative emotions are perceived as more aversive when preceded by a positive emotion [81]. Given the combination of heightened emotional and interpersonal sensitivity in BPD (including intense emotional suffering and fears of abandonment and rejection) [2, 75], individuals with elevated BPD symptoms may be particularly likely to experience affiliative emotions as threatening due to fears that the negative emotions that will inevitably replace these positive emotions will be experienced as even more aversive. Alternatively, this emotional and interpersonal sensitivity may interfere with the experience of positive emotions to such an extent that these emotions are so foreign that they are perceived as threatening and not able to be trusted [2]. Then again, given the greater overlap between fear of compassion from others (vs. for others) and fear of self-compassion [48, 49], as well as findings of a strong negative association between BPD pathology and self-compassion [82, 83], it may be that the fear of compassion from others (vs. for others) is simply more relevant to BPD pathology in general.

Results must be considered in light of limitations present. First, data were correlational and cross-sectional. Thus, although we were able to examine if fears of compassion from and for others accounted for significant variance in the relations of BPD symptoms to ineffective conflict resolution strategies, we cannot draw any conclusions about the temporal relations among these factors or the extent to which fears of compassion predict the use of ineffective conflict strategies. Indeed, although the proposed model is grounded in theoretical and empirical literature on BPD, fears of compassion, and conflict, the examined relations are likely complex and bidirectional. For example, repeated use of ineffective conflict resolution strategies could increase fears of compassion and/or exacerbate BPD symptoms. In the absence of prospective data, results of this study should be interpreted with caution and require replication in prospective longitudinal and micro-longitudinal (e.g., ecological momentary assessment) studies.

Additionally, our exclusive reliance on self-report measures introduces the potential for recall and social desirability biases. Thus, future studies would benefit from using clinician-administered interviews and behavioral observations of responses to conflict. Likewise, although the measure of conflict resolution strategies used in this study has good psychometric properties, there are limitations associated with assessing aspects of interpersonal relationships and interpersonal interactions among one member of a dyad only. Future studies should therefore include partner reports of conflict resolution strategies as well. In addition, laboratory-based studies of romantic partner dyads that assess responses to conflict in the laboratory would be particularly useful for examining more nuanced associations among BPD pathology, fears of compassion, and responses to romantic relationship conflict, as well as determining the effectiveness of the conflict resolution strategies employed. Finally, our study utilized a sample of patients in a community-based correctional SUD residential treatment facility. Given evidence that patients in residential SUD treatment facilities are characterized by more severe symptom presentations [84], findings from this study may not generalize to outpatient SUD samples or community adults with SUDs. Likewise, given that only one fifth of our sample identified as a member of a racial/ethnic minoritized group, findings may not generalize to individuals from more diverse racial/ethnic backgrounds. Thus, replication of these findings in other SUD samples, including more ethnically and racially diverse samples, is needed.

## Conclusions

Despite limitations, the results of this study add to the literature on the negative outcomes associated with BPD pathology among individuals with SUDs, highlighting the relevance of both BPD symptoms and fear of compassion from others to ineffective responses to romantic relationship conflict surrounding substance use among SUD patients. In particular, findings suggest that the fear of compassion from others explains the relation between BPD symptoms and ineffective conflict resolution strategies within this population. These findings highlight the potential utility of interventions aimed at reducing fears of compassion and increasing comfort with and tolerance of compassion from both others and oneself among SUD patients with BPD symptoms in order to strengthen relationships and reduce risk for relapse.

## Data Availability

The dataset of the current study is available from the corresponding author on reasonable request.

## Abbreviations

ASI: Addiction Severity Index
BPD: Borderline personality disorder
FOCS: Fear of Compassion Scale
MSI-BPD: McLean Screening Instrument for Borderline Personality Disorder
RPCS: Romantic Partner Conflict Scale
SUD: Substance use disorder
